# Cadmium Mimics Estrogen-Driven Cell Proliferation and Prolactin Secretion from Anterior Pituitary Cells

**DOI:** 10.1371/journal.pone.0081101

**Published:** 2013-11-13

**Authors:** Sonia A. Ronchetti, Eliana A. Miler, Beatriz H. Duvilanski, Jimena P. Cabilla

**Affiliations:** Instituto de Investigaciones Biomédicas (UBA-CONICET), Facultad de Medicina, Universidad de Buenos Aires, Buenos Aires, Argentina; Universidad Miguel Hernández de Elche, Spain

## Abstract

Cadmium (Cd) is a heavy metal of considerable occupational and environmental concern affecting wildlife and human health. Recent studies indicate that Cd, like other heavy metals, can mimic effects of 17β-estradiol (E2) involving E2 receptor (ER) activation. Lactotrophs, the most abundant cell type in anterior pituitary gland, are the main target of E2, which stimulates cell proliferation and increases prolactin secretion through ERα. The aim of this work was to examine whether Cd at nanomolar concentrations can induce cell proliferation and prolactin release in anterior pituitary cells in culture and whether these effects are mediated through ERs. Here we show that 10 nM Cd was able to stimulate lactotroph proliferation in anterior pituitary cell cultures from female Wistar rats and also in GH3 lactosomatotroph cell line. Proliferation of somatotrophs and gonadotrophs were not affected by Cd exposure. Cd promoted cell cycle progression by increasing cyclins D1, D3 and *c-fos* expression. Cd enhanced prolactin synthesis and secretion. Cd E2-like effects were blocked by the pure ERs antagonist ICI 182,780 supporting that Cd acts through ERs. Further, both Cd and E2 augmented full-length ERαexpression and its 46 kDa-splicing variant. In addition, when co-incubated Cd was shown to interact with E2 by inducing ERα mRNA expression which indicates an additive effect between them. This study shows for the first time that Cd at nanomolar concentration displays xenoestrogenic activities by inducing cell growth and stimulating prolactin secretion from anterior pituitary cells in an ERs-dependent manner. Cd acting as a potent xenoestrogen can play a key role in the aetiology of different pathologies of the anterior pituitary and in estrogen-responsive tissues which represent considerable risk to human health.

## Introduction

Cadmium (Cd) is a heavy metal that is dispersed throughout the environment mainly as a result of pollution from industrial and agricultural practices [1,2]. Asides from occupational exposure, human intoxication results from consumption of contaminated water and food or inhalation of cigarette smoke [3]. Since Cd can not be degraded, the risk of environmental exposure and contamination is constantly increasing because of accumulation via both water and the food chain [2] and also Cd long half-life (over 26 years) in the whole body in humans.

The reproductive health of humans and wild animals has progressively deteriorated in the last 50 years [4]. It has been suggested that environmental endocrine disruptors may play a role in the aetiology of this pathology since the hypothalamic–pituitary–gonadal axis is a target for many toxicants. Endocrine disrupting chemicals (EDCs) are natural or synthetic compounds that interfere in the biosynthesis, metabolism or action of endogenous hormones. A particular class of EDCs, called xenoestrogens (XEs), appears to trigger cell responses normally induced by estrogens and therefore, thereby affecting their signaling. Many chemicals in the environment can act as endocrine active compounds [5]. Several reports show that Cd possesses estrogen-like activity [6-9]. In the last decade, Cd has also been shown to have potent estrogen- and androgen-like activities *in vivo* and *in vitro* by directly binding to estrogen and androgen receptors [10-12]. 

The major female hormone, 17β-estradiol (E2), is a key regulator of pituitary physiology involved in hormone release as well as proliferation and cell death in anterior pituitary gland [13,14]. E2 exerts its effects through activation of multiple genomic and non genomic signal pathways. Estrogen actions are mediated by two specific intracellular estrogen receptors (ERs), ERα and ERβ, belonging to the steroid/thyroid hormone superfamily of transcription factors [15]. Genomic signaling takes place when ligands enter the cell and bind ER to induce dimerization. ER dimers act as hormone-dependent transcription regulators by directly binding DNA at estrogen responsive elements (ERE) sequences or indirectly by tethering to DNA through other transcriptions factors like Sp1 or AP-1 [16]. Non-genomic E2 actions involves rapid activation of membrane-associated ERs which triggers second-messenger signaling. This pathway mediates some E2 rapid actions such as activation of nitric oxide synthesis and actin cytoskeleton remodeling. Membrane-iniciated E2 actions are not fully understood yet. To date, little is known about non-genomic–dependent proliferation and hormone secretion. E2 stimulatory effects on prolactin secretion and lactotroph proliferation are mediated by ERα Three forms of ERα have been reported: the full-length 66 kDa ERα isoform (ERα66) and two truncated splice variants (truncated estrogen receptor products or TERPs) of 36 kDa (ERα36 or TERP1) and 46 kDa (ERα46 or TERP2). These splice variants have been detected first in the pituitary gland and then in other tissues including breast, endometrium, smooth muscle cells and peripheral blood mononuclear cells [17,18]. 

Anterior pituitary gland consists of several cell types essential for many physiological processes such as growth, development, homeostasis, metabolism, and reproduction. Almost 50% of the gland is constituted by lactotrophs, which secrete prolactin and, together with gonadotrophs, are the main target of E2 actions on anterior pituitary. 

Estrogens are one of the major regulators of anterior pituitary physiology, eliciting a plethora of processes, including the stimulation of lactotroph proliferation and up-regulation of prolactin gene expression, synthesis, storage and release. 

E2 regulation of target cell proliferation results from promotion of both cell growth and survival [19]. This hormone controls the function of several genes and proteins directly involved in cell cycle regulation, including cyclins, proto-oncogenes, and negative cell cycle regulators. Progression through the cell cycle is strictly regulated by different proteins such cyclins (A, B, D and E) associated to cyclin-dependent kinases [20]. Several lines of evidence have linked estrogen regulation of cell proliferation to cyclin D1 expression [21].

*c-fos* and *c-jun* proto-oncogenes are members of the AP-1 transcription factor that are rapidly induced by mitogenic stimuli [22]. E2 was shown to stimulate *c-fos* expression in lactotrophs and follicle-stellate cells [23]. 

Cd has been proposed to alter anterior pituitary secretion through feedback mechanisms or by directly affecting anterior pituitary cells [24,25]. Previous results from our group have shown that Cd at micromolar concentrations induces oxidative stress and decreases prolactin release both *in vivo* and *in vitro* [3,26,27]. At the same time, Cd at nanomolar concentrations displays estrogen-like activities in several E2-responsive cell lines [7,28].

Most studies of Cd xenoestrogenic effects have been performed on cell lines derived from different tumors. It has been reported that Cd like E2 is able to stimulate cell proliferation and induce the expression of E2-responsive genes in MCF-7 breast cancer cell line [6,8]. Bearing in mind the adverse effect of Cd on anterior pituitary and the role of this gland in reproductive and normal endocrine function, the aim of this study was to investigate whether Cd has estrogen-like activities on anterior pituitary cells by addressing Cd effects on anterior pituitary cell proliferation and prolactin secretion. 

## Materials and Methods

### Ethics Statement

All experimental procedures were approved by the Committee on Ethics of the School of Medicine (University of Buenos Aires, Res. (CD) No. 2831/10) and were conducted in compliance with the guidelines of the NIH Guide for the Care and Use of Laboratory Animals.

### Materials

CdCl_2_ (Cd) was purchased from Mallinckrodt Chemical Works (St. Louis, MO, USA). Go Taq DNA polymerase, random hexamers and dNTPs were provided by Promega (Madison, WI, USA). TRIzol and molecular biology reagents were from Invitrogen (Carlsbad, CA, USA). Media and reagents for cell culture were purchased from Gibco (Rockville, MD, USA) except for the fetal bovine serum and horse serum that were obtained from GEN SA (Buenos Aires, Argentina). Cyclin D1 and ERα antibodies were purchased from Santa Cruz Biotechnology (Santa Cruz, CA, USA). Anti-recombinant rat prolactin antiserum was provided by Dr. A. F. Parlow, National Hormone and Pituitary Program, Torrance, CA, USA. Unless otherwise indicated, all other reagents and antibodies were obtained from Sigma-Aldrich (St. Louis, MO, USA).

### Animals

Adult female Wistar rats (180-200 g) were used at random stages of estrous cycle. Animals were kept in controlled conditions of light (12:12 h light/dark cycle) and temperature (21-24 °C). Food and water were supplied *ad libitum*. 

### Cell culture

Animals (8-10 per experiment) were killed by decapitation and anterior pituitary glands without neural lobe were removed. Cells were obtained from pooled anterior pituitary glands by enzymatic (trypsin/DNase) and mechanical dispersion (extrusion through a Pasteur pipette) as previously described [29]. Cell viability was assessed by the trypan blue exclusion method. In all cases, viability was greater than 90%. Dispersed cells were seeded onto tissue culture plates and stabilized for 24 h (37 °C, 5% CO_2_ in air) in phenol red-free Dulbecco’s modified Eagle’s medium (DMEM) supplemented with 10% charcoal-stripped fetal bovine serum (CSFBS), 10 µL/mL MEM amino acids, 2 mM glutamine, 5.6 µg/mL amphotericin B and 25 µg/mL gentamicin (DMEM-S-10% CSFBS). For immunocytochemistry experiments, cells were seeded on glass coverslips in 24-well tissue culture plates (1.10^5^ cells/well). For mRNA and protein expression experiments, cells were seeded in 6-well tissue culture plates (2.10^6^ cells/well).

### Cell treatment

After the stabilization period (24 h), cells were synchronized in free serum media for 24 h. Subsequently, they were incubated for different times (8 to 96 h) with vehicle (control) or 10 nM Cd, with or without 100 nM ICI 182,780, an antagonist of estrogen receptors (added 30 min before the treatments). E2 (1 nM) was used as positive control of the experiments. 

### GH3 cell culture

GH3 cells were cultured in F-12K Nutrient Mixture (Kaighn's modification, GIBCO) supplemented with 15% horse serum, 2.5% fetal bovine serum, 1% penicillin/streptomycin and 0.1% amphotericin B (pH 7.35; 37 °C, 5% CO_2_ in air). The incubation medium was changed every 2 days. Cells were harvested once per week by treatment with phosphate-buffered saline containing trypsin (2.5 mg/mL; GIBCO), and reseeding was carried out at 20% of the original density. 

### GH3 cells treatment

Cells were tripsinized, washed three times in serum free media and seeded in DMEM-S-10% CSFBS. After the stabilization period (24 h), cells were synchronized in free serum media for 24 h. Cells were incubated with vehicle (control) or 10 nM Cd for different times. E2 (1 nM) was used as positive control of the experiments.

### Immunocytochemistry (ICC)

Anterior pituitary cultures were incubated with 100 μM BrdU 24 h before the end of the treatment. GH3 cells were incubated with 10μM BrdU 3 h before the end of the treatment. Cells were fixed in 4% formaldehyde for 30 min at 4 °C, permeabilized with 6N HCl in 1% Triton X-100 in PBS for 15 min at room temperature and neutralized with 0.1 M sodium borate in 1% Triton X-100 in PBS for 15 min at room temperature. Then, cells were incubated in blocking solution (5% normal serum in 0.2% Triton X-100) for 2 h at room temperature. Cells were incubated with mouse anti-BrdU primary antibody (1:200) overnight at 4 °C and after three washes the secondary antibody conjugated to fluorescein (1:250) was added. Cells were mounted in anti-fade solution containing DAPI and DABCO. Cells were observed and quantified in an Olympus BX50 (Japan) fluorescence microscope. Data of at least 300 nuclei per triplicate obtained from random fields and from three independent experiments were expressed as number of BrdU-labeled cells / total cell number x 100. 

After BrdU labeling, cells were incubated with guinea pig primary antibodies (PRL, 1:2500; GH, 1:2000; LH: 1:2500) and mouse anti-BrdU antibody (1:200) overnight at 4 °C. Then cells were washed and incubated with secondary antibodies for 2 h at 37 °C. Cells were mounted and counted as described above.

### RNA isolation

177 μL of TRIzol reagent was added to each well. After isolation, total RNA from tissues was spectrophotometrically quantified at 260 nm. RNA integrity was checked by formaldehyde/formamide gel electrophoresis. 

### RT and PCR reactions

First strand cDNA was synthesized with Moloney murine leukemia virus (M-MLV) reverse transcriptase in buffer containing 5.5 mM MgCl_2_, 0.5 mM dNTP, 2.5 μM random hexamers, and 3.125 U/μL M-MLV reverse transcriptase. Reactions were done in a final volume of 12 μL containing 1 μg RNA. The reverse transcription reaction was run at 37 °C for 50 min and reverse transcriptase was inactivated by heating the samples at 70 °C for 15 min before the PCR reactions. To check for genomic contamination, the same procedure was used on samples in a reaction solution lacking reverse transcriptase.

Specific primers for the genes studied were checked with Oligo Perfect designer software (Invitrogen) as detailed in Table **1**. GAPDH was used as endogenous control. Then, samples were thermocycled for PCR amplification (Mastercycler, Eppendorf, Hamburg, Germany). The reaction mixture contained GoTaq PCR buffer, 1.5 mM MgCl_2_, 200 μM of each dNTP, 0.625 U GoTaq polymerase and 300 nM of each primer. We utilized RT-PCR methods to determine relative changes in mRNA expression. Cycles of PCR amplification are detailed in Table **2**. Amplified products collected from various cycles were analyzed by electrophoresis in 2% agarose-ethidium bromide gels.

**Table 1:** Primers used for semi-quantitative RT-PCR assays.

**Gene**	**Primer**		**Product size (bp)**
**Cyc D1**	Forward	5' CGCCCTCCGTTTCTTACTTCA 3'	255
**Cyc D1**	Reverse	5' AACTTCTCGGCAGTCAGGGGA 3'	
**Cyc D3**	Forward	5' GCGTCCCCACCCGAAAGGCG 3'	386
**Cyc D3**	Reverse	5' TAGAGCAGGCACCCAGGCCT 3'	
ERα	Forward	5' TCCACGATCAAGTTCACC 3'	311
ERα	Reverse	5' GGATGTGGTCCTTCTCTT 3'	
**PRL**	Forward	5' AGCCAAGTGTCAGCCCGGAAAG 3'	237
**PRL**	Reverse	5' TGGCCTTGGCAATAAACTCACGA 3'	
***c-fos***	Forward	5' CCAACTTTATCCCCACGGTGAC 3'	381
***c-fos***	Reverse	5' TGGCAATCTCGGTCTGCAAC 3'	
**GAPDH**	Forward	5' TGCACCACCAACTGCTTA 3'	176
**GAPDH**	Reverse	5' GGATGCAGGGATGATGTTC 3'	

**Table 2:** PCR amplification schemes.

**Gene**	**Cycle number**	**Scheme**
		1 min, 94 °C
**Cyc D1 and D3**	50 (cyc D1)	1 min, 60 °C
	38 (cyc D3)	2 min, 72 °C
		30 sec, 95 °C
ERα	40	45 sec, 55 °C
		40 sec, 72 °C
		30 sec, 95 °C
**PRL**	38	60 sec, 56 °C
		45 sec, 72 °C
		30 sec, 94 °C
***c-fos***	42	30 sec, 60 °C
		45 sec, 74 °C

### Analysis of semi-quantitative PCR data

The intensity of PCR products was determined by digital image analysis using Gel Pro Analyzer (Media Cybernetics, LP, Silver Spring, MD) software for Windows. For statistical comparison of results from different experiments, data were normalized to the value of the GAPDH amplified band in each lane.

### Preparation of cell homogenates for immunoblot analysis

Anterior pituitary cells in culture were tripsinized and sonicated in lysis buffer containing 50 mM HEPES pH 7.4, 150 mM NaCl, 1 mM EDTA, 0.1% SDS, 10 µg/mL leupeptin, 10 µg/mL pepstatin and 1 mM PMSF. Homogenates were centrifuged for 20 min at 10,000 x *g* (4 °C) and the post-mitochondrial fraction was used in the immunoblot analysis.

### Protein measurement

Protein content of supernatants was measured by Bradford reagent, using bovine serum-albumin as standard.

### Immunoblot analysis

Fifty to eighty micrograms of total protein from each sample was boiled for 5 min in Laemmli sample buffer and fractioned on 10% SDS-PAGE. Resolved proteins were transferred to polyvinylidene difluoride membranes and blocked for 2 days at 4 °C in blocking buffer (5% nonfat dry milk in 1% PBS). Then, membranes were co-incubated overnight at 4 °C with rabbit antisera anti-Cyc D1 (1:500) or ERα (1:500) or prolactin (1:100000) together with anti-actin (1:1000) in blocking buffer. Blots were washed and incubated for 1 h at room temperature with horseradish-peroxidase conjugated goat antirabbit IgG (1:2000), followed by detection of immunoreactivity with diaminobenzidine solution containing 0.01% hydrogen peroxide.

### Analysis of immunoblot data

The intensity of immunoblot signals was determined by digital image analysis using Gel Pro Analyzer software for Windows. For statistical comparison of results from different blots, levels were normalized to the value of the actin immunoreactive band in each lane*.*

### Hormone determination

Prolactin (PRL) was measured by a double antibody radioimmunoassay (RIA) [30] using reagents provided by Dr. A.F. Parlow (National Hormone and Pituitary Program, Torrance, CA, USA). Recombinant prolactin (NIDDK-rPRL-RP-3) was used as reference preparation and NIDDK-antirPRL-S-9 as antiserum. The sensitivity of the assay was 0.1 ng/mL. Intra- and inter-assay coefficients of variation were under 10%. To avoid inter-assay variations, all samples were measured in the same assay. Prolactin level in control media at 8 h was 2825 ng/mL. 

### Statistical analysis

Results are expressed as mean ± SE and evaluated by one-way ANOVA followed by Tukey’s, Dunnett’s or Student’s ‘t’ test, depending on the experimental design. Differences between groups were considered significant if p<0.05. Results were confirmed by at least three independent experiments.

## Results

### Cadmium stimulates anterior pituitary cell proliferation

Many authors have reported that E2 stimulates anterior pituitary cell proliferation. To determine whether Cd affects proliferation of these cells, primary cultures of anterior pituitary cells were incubated with increasing concentrations of Cd ranging from 10^-12^ M to 10^-6^ M for 96 h. Cell proliferation was determined by BrdU incorporation into DNA. Since a significant increase of cell proliferation was observed with 10 nM Cd treatment, this concentration was chosen for subsequent studies. The growth rate induced by 10 nM Cd was very similar to that observed with 1 nM E2 (BrdU incorporation %; Control: 23.8 ± 2.2; Cd: 45.5 ± 2.7**; E2: 45.5 ±4.8**; N=3. ANOVA followed by Dunnett’s test, **p<0.01 vs. control). 

### Cadmium stimulates lactotroph proliferation

Anterior pituitary gland is constituted by a heterogeneous secretory cell population, of which lactotrophs are one of the main targets of E2 action. 

**Figure 1:**
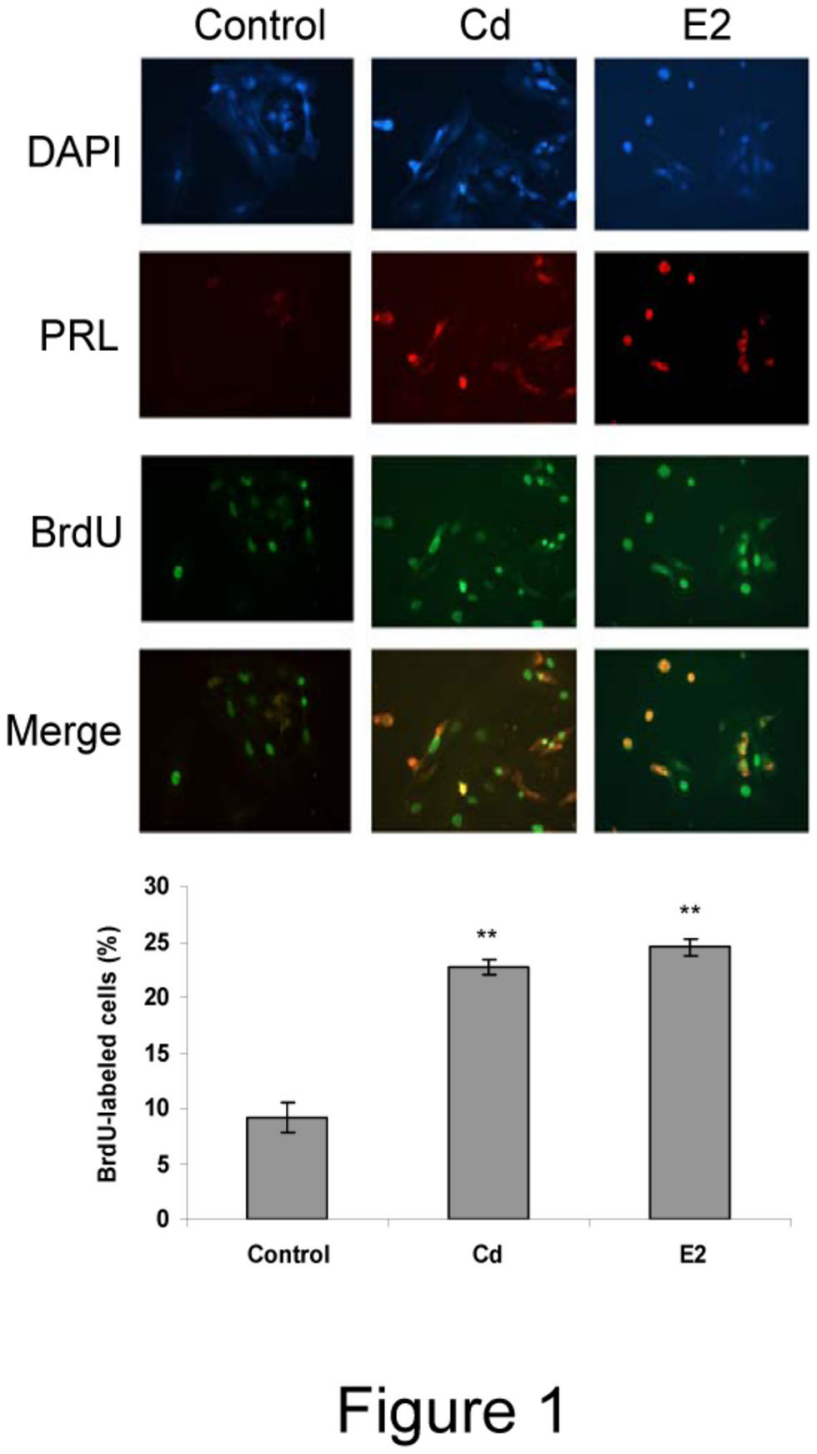
Cadmium stimulates anterior pituitary lactotroph proliferation. Anterior pituitary cells were treated with vehicle (control), 10 nM Cd or 1 nM E2 for 96 h. Cell growth was determined by ICC measuring 24 h-BrdU incorporation. Lactotrophs were identified by prolactin-specific antibody and cell nuclei were stained by DAPI. Pictures are representative of three independent experiments performed in triplicate. Bars represent the mean ± SE of BrdU-labeling index expressed as positive BrdU lactrotroph / total lactotroph cell number x100. ANOVA followed by Tukey-Kramer’s test, **p<0.001 vs. control (N=3).

To identify which pituitary cell type is affected by Cd proliferative effects, specific antibodies against different pituitary hormones such as PRL, GH and LH were used followed by BrdU incorporation and DAPI staining. The ICC study revealed that only lactotrophs showed an increase in BrdU incorporation after 96 h of Cd treatment (Figure **1**). Somatotroph and gonadotroph cell numbers were unaffected by either Cd or E2 exposure (data not shown). 

### Cadmium stimulates cyclin (Cyc) D1 and D3 and c-fos gene expression

**Figure 2:**
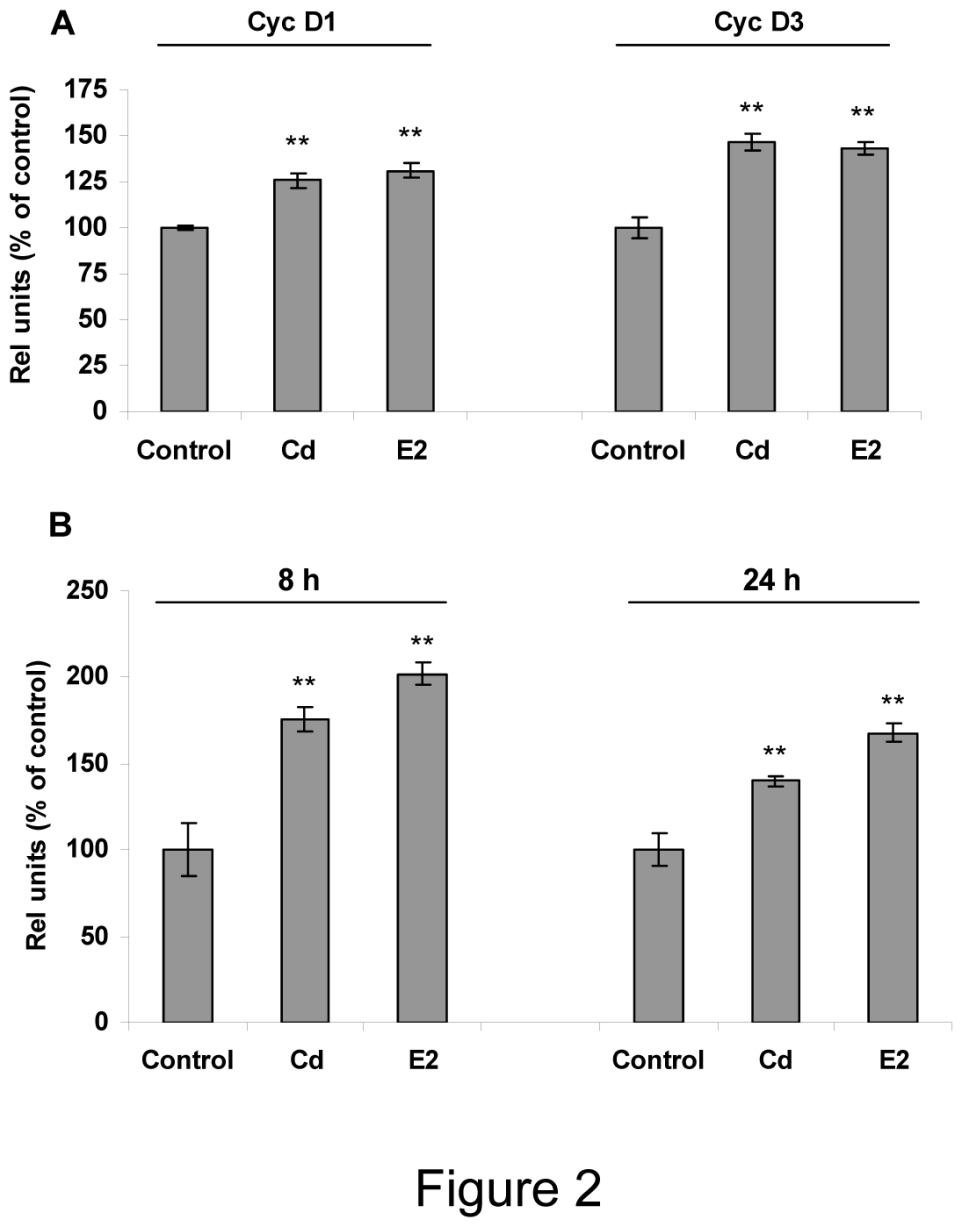
Cadmium increases gene expression of proliferation markers in anterior pituitary cells. Anterior pituitary cells were treated with vehicle (control), 10 nM Cd or 1 nM E2. Gene expression was evaluated by PCR. Bars represent the mean ± SE of densitometric values of cyclins D1 and D3 after 72 h (A) or *c-fos* after 8-24 h (B) normalized to GAPDH expression and are expressed as percent of control. ANOVA followed by Tukey-Kramer’s test, *p<0.05, **p<0.001 vs. control (N=3).

**Figure 3:**
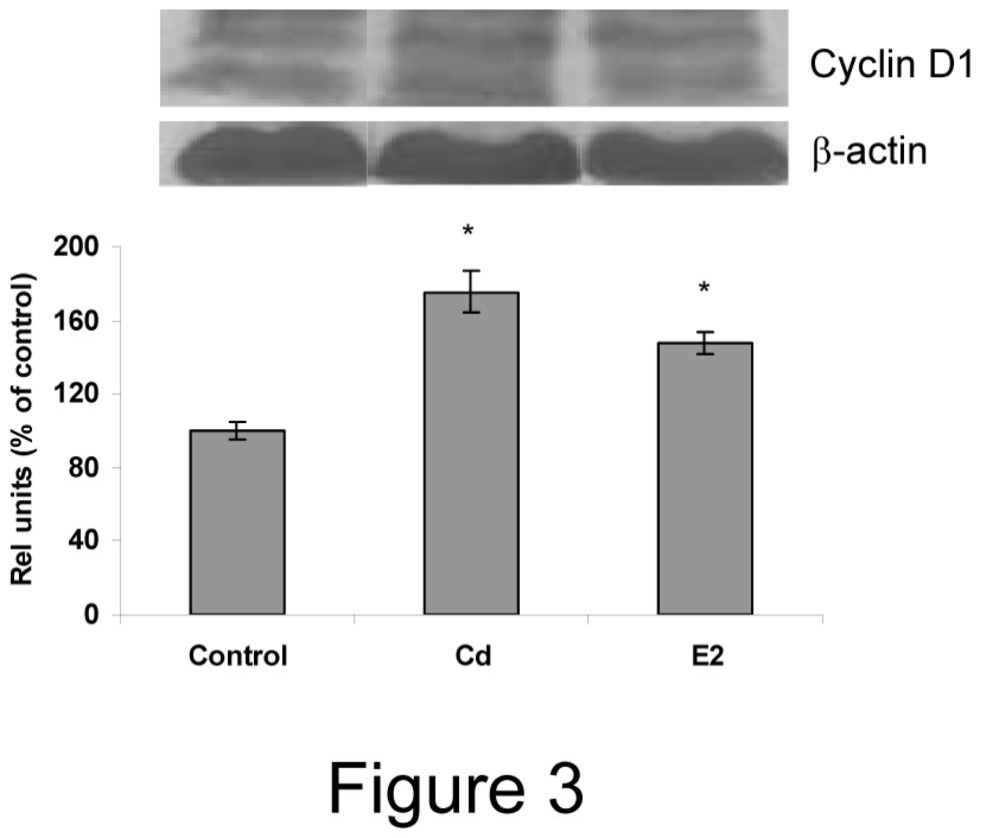
Cadmium increases cyclin D1 protein expression in anterior pituitary cells. Anterior pituitary cells were treated with vehicle (control), 10 nM Cd or 1 nM E2 for 72 h. A representative western blot is shown. Bars represent the mean ± SE of densitometric values normalized to β-actin expression and are expressed as percent of control. ANOVA followed by Tukey-Kramer’s test, *p<0.05 vs. control (N=3).

Cyclins and proto-oncogenes are directly involved in cell cycle progression. To further confirm Cd proliferative effects in anterior pituitary cells, we examined whether this metal is able to induce Cyc D1, Cyc D3 and c-fos gene expression. Anterior pituitary cultures were incubated with 10 nM Cd or 1 nM E2, then mRNA levels of c-fos and Cyc D1 and D3 were determined after 8-24 h or 72 h, respectively. Both Cd and E2 treatments were able to similarly increase Cyc D1, Cyc D3 (Figure **2A**) and c-fos mRNA levels (Figure **2B**). Cyc D1 protein expression was also determined by western blot. Since the antibody used is strongly cross-reactive with cyclin D2 and D3, both of lower molecular weight, we only analysed the upper band (approximately 38 kDa) corresponding to cyclin D1. We observed a significant increase of cyclin D1 expression after both Cd and E2 treatment (Figure **3**). These results suggest that Cd is able to reproduce E2 proliferative effects in anterior pituitary cells.

### Cadmium stimulates GH3 cell proliferation and Cyc D expression

GH3 is a lactosomatotroph cell line derived from a rat E2-responsive pituitary tumor. To further confirm the specific Cd xenoestrogenic effect on lactotroph proliferation, we studied Cd effects on GH3 cell proliferation. 

**Figure 4:**
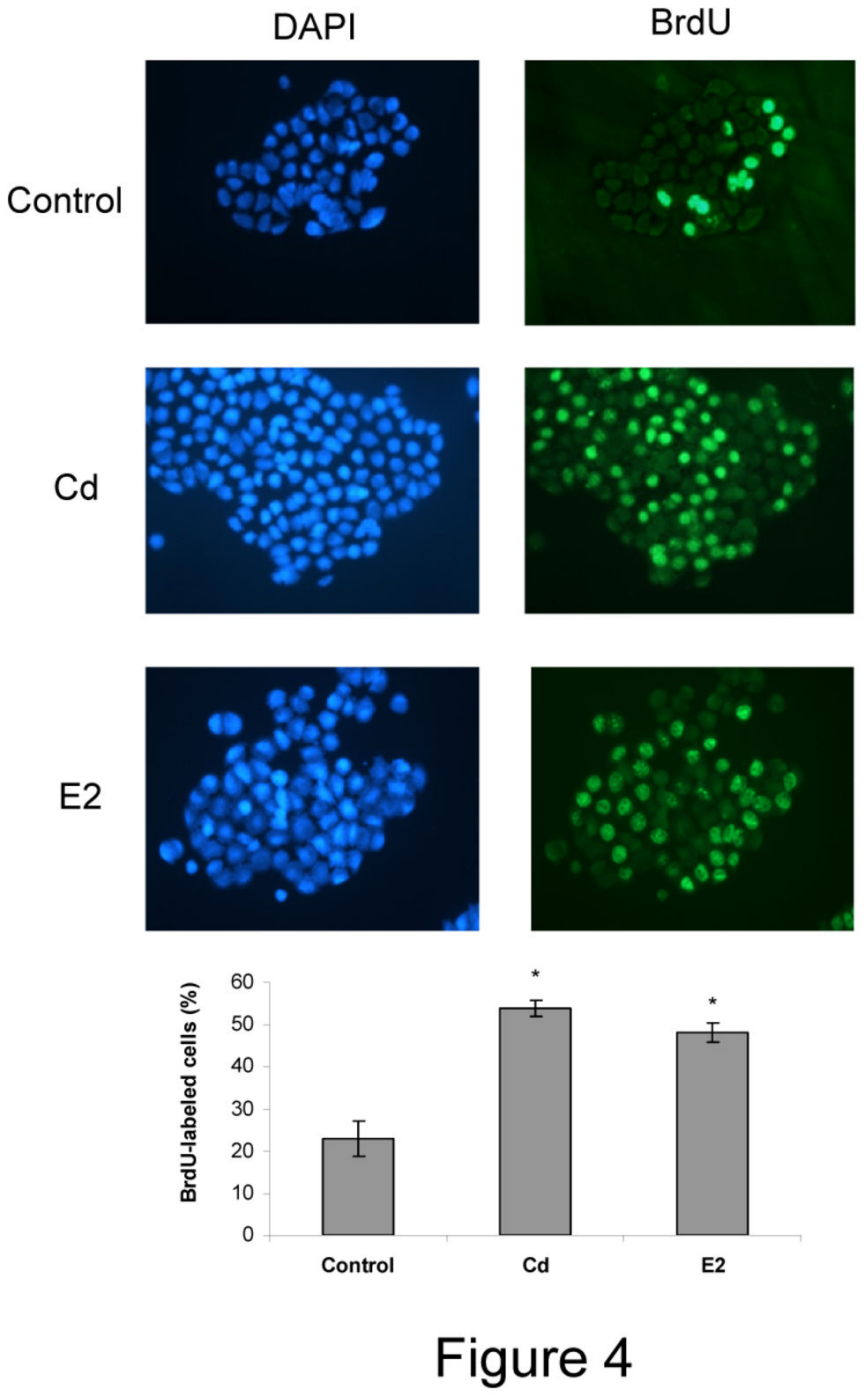
Cadmium stimulates GH3 cell proliferation. GH3 cells were treated with vehicle (control), 10 nM Cd or 1 nM E2 for 96 h. Cell growth was determined by ICC measuring 3 h-BrdU incorporation. Cell nuclei were stained by DAPI. Pictures are representative of three independent experiments performed in triplicate. Bars represent the mean ± SE of BrdU-labeling index expressed as positive BrdU cells / total cell number x 100. ANOVA followed by Tukey-Kramer’s test, *p<0.05 vs. control (N=3).

After GH3 cells were incubated with 10 nM Cd or 1 nM E2, cell proliferation and Cyc D1 and D3 mRNA expression were measured. Cd treatment, like E2, was able to significantly stimulate BrdU incorporation (Figure **4**) and cyclin mRNA levels (relative units as percent of control: Cyclin D1, control: 100±8.9; Cd: 135.4±2.6*; E2: 136±3.3*. Cyclin D3, control: 100±4.5; Cd: 118.3±1.6*; E2: 118.4±3.4*. ANOVA followed by Tukey-Kramer’s test, *p<0.05 vs. control). 

#### Cadmium increases prolactin synthesis and secretion

**Figure 5:**
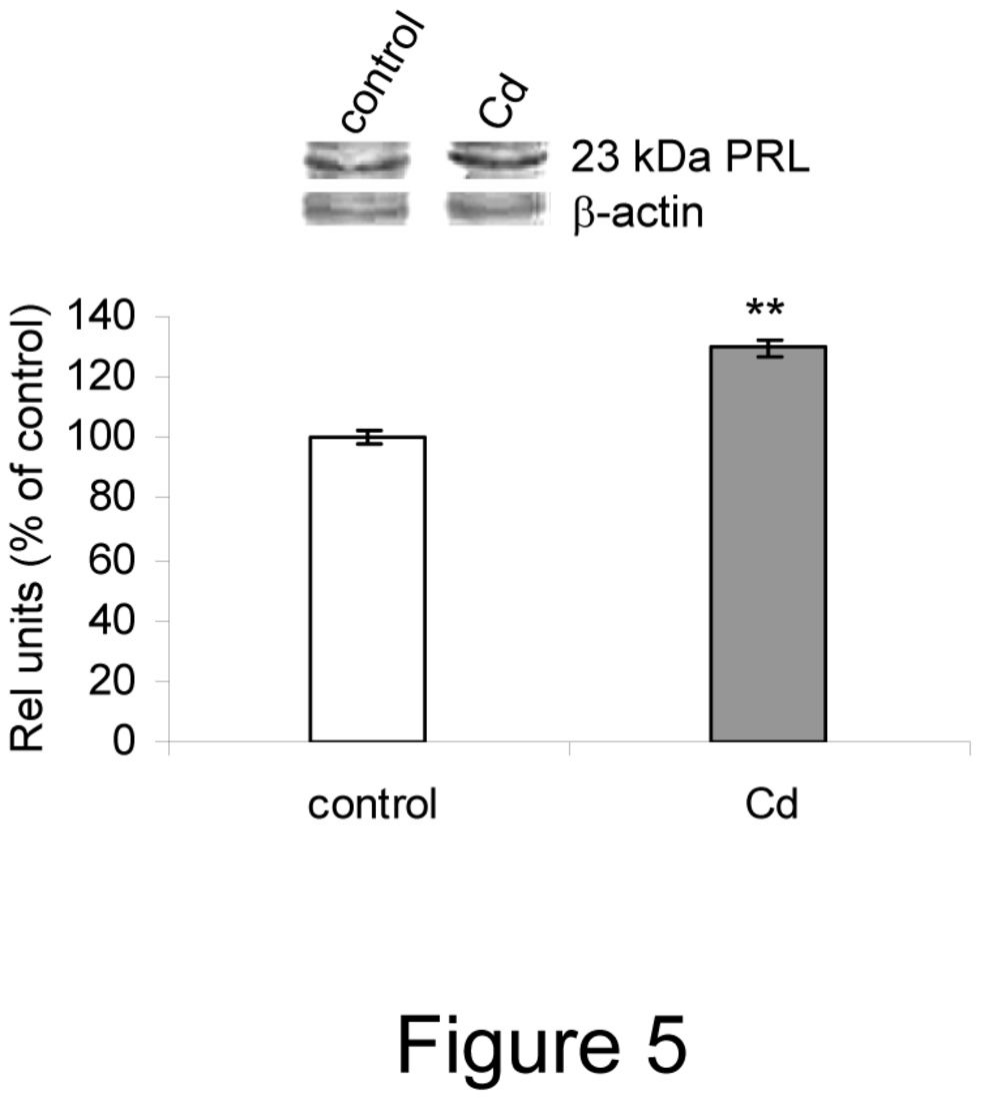
Cadmium increased 23 kDa prolactin (PRL) protein expression in anterior pituitary cells in culture. Anterior pituitary cells were incubated with 10 nM Cd or vehicle (control) for 8 h. Protein expression was measured by western blot. Bars represent mean ± SEM of PRL densitometric values normalized to β-actin and are expressed as percent of control. **P<0.01, Student’s ‘t’ test (N=3).

The stimulatory effect of E2 on prolactin secretion is well known. To examine whether Cd mimics E2 action on both prolactin synthesis and release, we measured prolactin mRNA and protein expression and hormone levels in culture media by PCR, western blot and RIA, respectively. Cd treatment increased prolactin mRNA levels (Table **3**), protein expression (Figure 5) and hormone release over the times studied (PRL release, fold of increase over respective control; 8 h: 1.5, 24 h: 3.0, 72 h: 4.4). Cd seems to affect prolactin release in a time-dependent manner.

**Table 3:** Cadmium stimulates prolactin mRNA expression.

	**Rel units (% of control)**	
	**Control**	**Cd**
**8 h**	100.0 ± 5.2	169.9 ± 18.3*
**24 h**	100.0 ± 9.0	180.3 ± 15.5**
**72 h**	100.0 ± 9.7	192.4 ± 13.2**

Anterior pituitary cells were treated with vehicle (control) or 10 nM Cd for 8, 24 or 72 h. Prolactin mRNA was determined by PCR. Densitometric values were normalized to GAPDH expression and are expressed as mean ± SE. ANOVA followed by Tukey-Kramer’s test, *p<0.05, **p<0.01 vs. respective control.

### Estrogen receptor (ER) mediates cadmium effect on Cyc D and PRL expression in anterior pituitary cells

**Figure 6:**
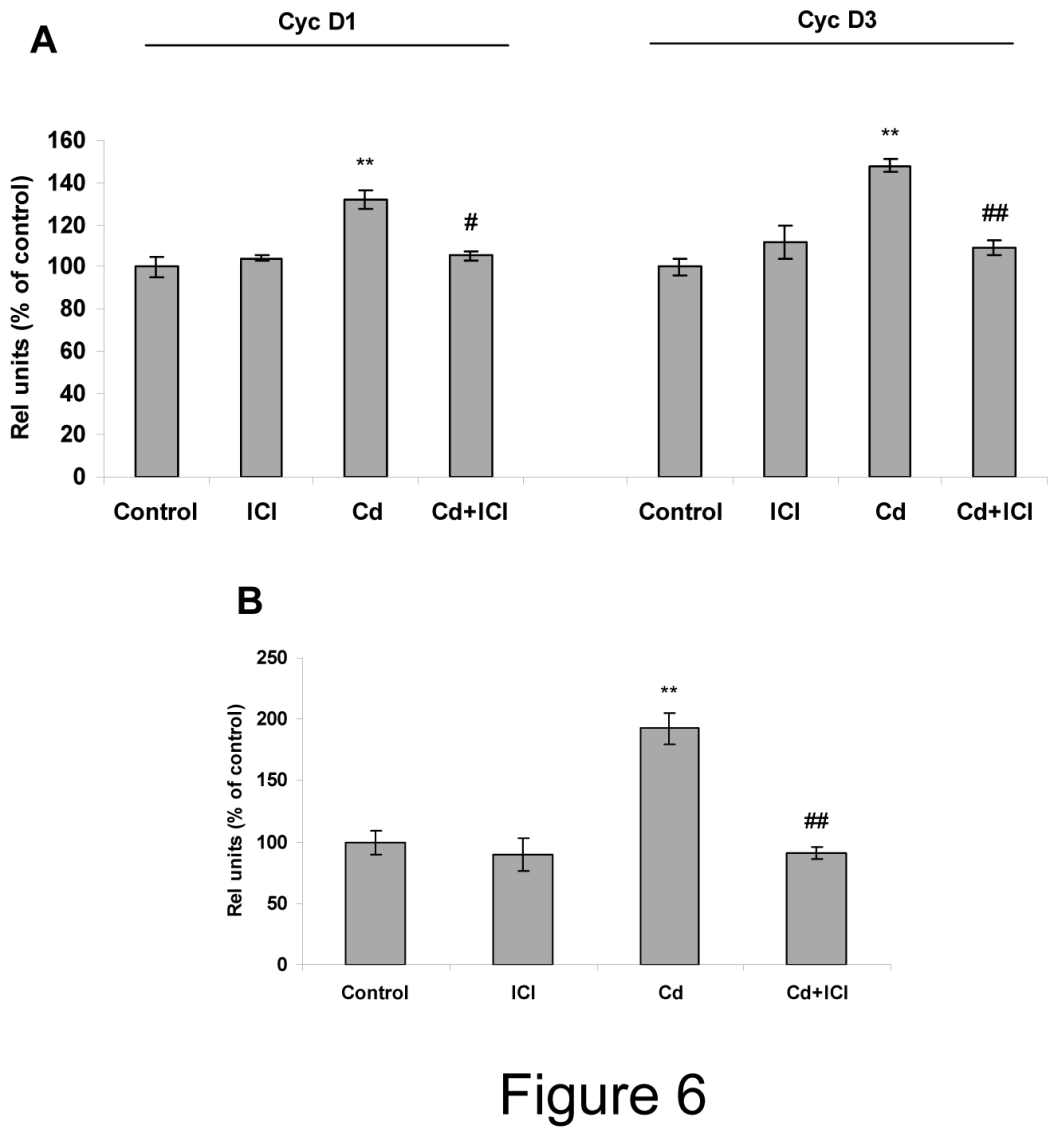
E2 receptors (ERs) mediates cadmium effect on cyclins D1 and D3 and PRL mRNA expression. Anterior pituitary cells cultures were first incubated with 100 nM ICI 182,780 (ICI) for 20 min and then incubated with vehicle (control) or 10 nM Cd for 72 h. Cyc D1 and D3 (A) and PRL (B) mRNA expression was evaluated by PCR. Bars represent the mean ± SE of densitometric values normalized to GAPDH and are expressed as percent of control. ANOVA followed by Tukey-Kramer’s test, **p<0.01 vs. control, ##p<0.01 vs. Cd (N=3).

E2/ERα pathway mediates cell proliferation and PRL secretion in anterior pituitary gland. To determine whether the effects of Cd were mediated by ERs, the ability of the pure ERs antagonist ICI 182,780 (ICI) to block E2-like Cd effects was tested. Anterior pituitary cells were first incubated with 100 nM ICI for 20 min followed by incubation with or without Cd for 72 h. Expression of Cyc D1, D3 and prolactin mRNA was evaluated by PCR. The antagonist had no effect by itself, but when co-incubated with Cd, it was able to completely block Cd effects on Cyc D1 and D3 (Figure **6A**) and PRL mRNA levels (Figure **6B**) suggesting that Cd effects are mediated by ERs. 

### Cadmium modifies ERαexpression in anterior pituitary cells

A variety of evidence indicates that E2 regulates the steady state levels of ERα protein and mRNA. To examine Cd effects on ERα expression, mRNA and protein levels were evaluated by PCR and western blot, respectively. 

**Figure 7:**
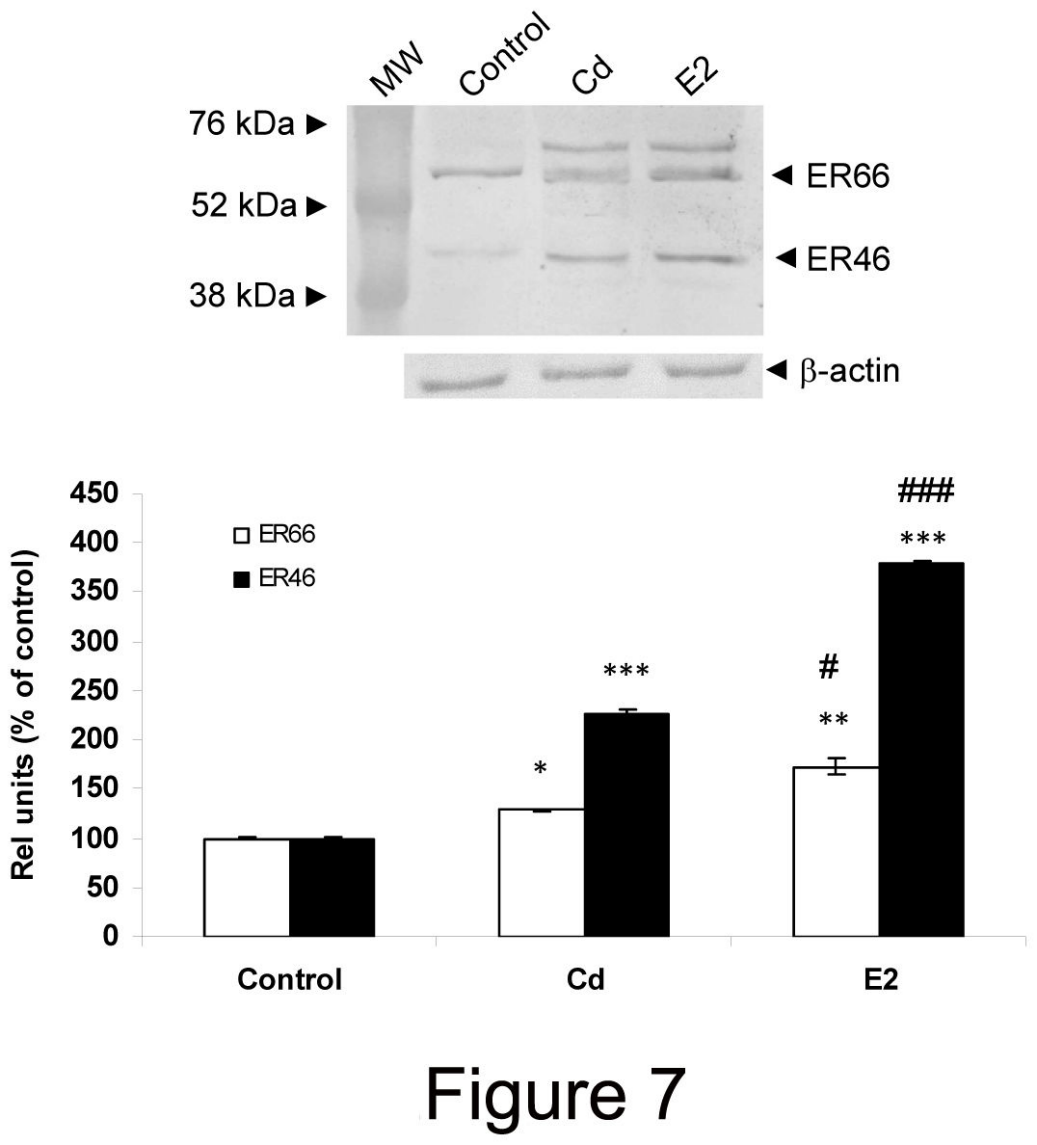
Cadmium exposure increases ERα **protein expression in anterior pituitary cells**. Anterior pituitary cells were treated with vehicle (control), 10 nM Cd or 1 nM E2 for 24h. A representative western blot is shown. Bars represent the mean ± SE of densitometric values of full-length ERα (open bars) and ERα46 (black bars) normalized to β-actin expression and are expressed as percent of control. ANOVA followed by Tukey-Kramer’s test, *p<0.05, **p<0.01, ***p<0.001 vs. respective control; #p<0.05, ###p<0.001 vs. Cd (N=3).

Cd exposure enhanced ERα mRNA expression in anterior pituitary cells at 8 and 24 h (Table **4**). In parallel, Cd and E2 increased full-length ERα66 and the splicing variant ERα46 at 8 h (data not shown) and after 24 h of exposure (Figure **7**). The upper, unidentified immunoreactive bands were discarded. In the same way, the splicing variant ERα36 was not included in this analysis because it was only detected after E2 treatment. 

**Table 4:** Cadmium modifies ERα mRNA expression in anterior pituitary cells.

	**Rel units (% of control)**	
	**Control**	**Cd**
**8 h**	100.0 ± 3.5	138.7± 9.6*
**24 h**	100.0 ± 5.1	141.6 ±2.0**

Anterior pituitary cells were treated with vehicle (control) or 10 nM Cd for 8 or 24 h. ERα mRNA was determined by PCR. Densitometric values were normalized to GAPDH expression and are expressed as mean ± SE. ANOVA followed by Tukey-Kramer’s test, *p<0.05, **p<0.01 vs. respective control.

### Cadmium and E2 co-treatment has an additive effect on ERα mRNA expression in anterior pituitary cells

**Figure 8:**
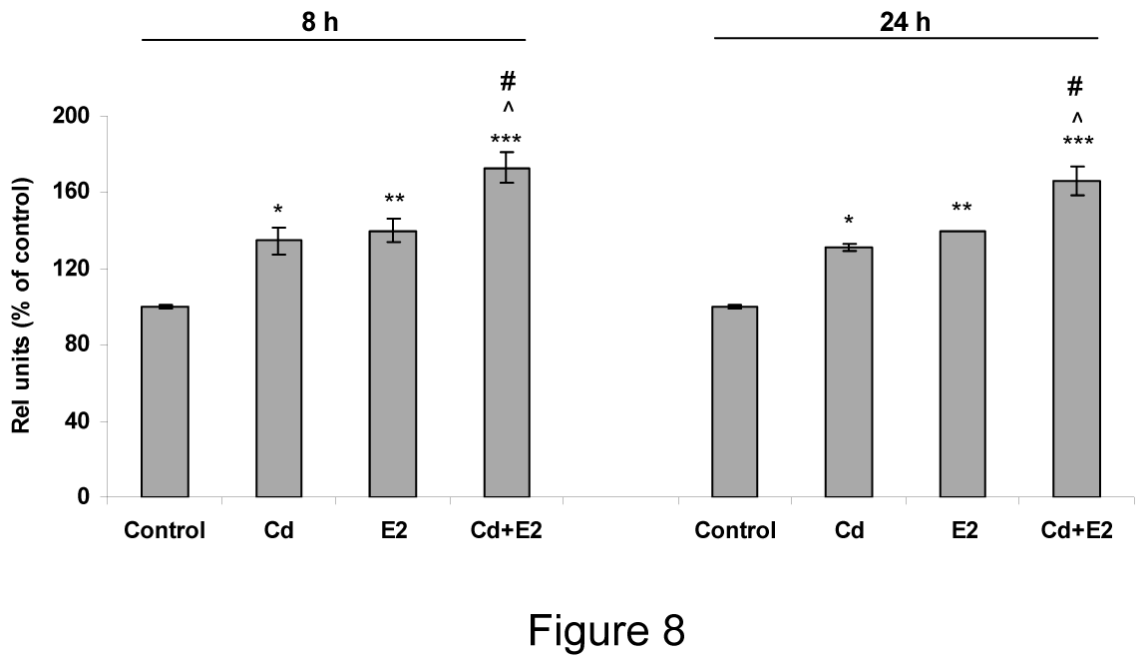
Additive effect of cadmium and E2 co-treatment on ERα mRNA expression. Anterior pituitary cell cultures were treated with vehicle (control), 10 nM Cd or 1 nM E2 or 10 nM Cd plus 1 nM E2 for 8 h or 24 h. ERα mRNA expression was evaluated by PCR. Bars represent the mean ± SE of densitometric values normalized to GAPDH. ANOVA followed by Tukey-Kramer’s test, *p<0.05, ** p<0.01, *** p<0.001 vs. Control; #p<0.05 vs. Cd; **^**p<0.05 vs. E2 (N=3).

Although there is evidence of interaction between Cd and ERα Cd effects in the presence of E2 have been poorly studied. To evaluate this condition, anterior pituitary cells were incubated for 8 and 24 h with Cd, E2 or Cd plus E2, then ERαmRNA expression was determined by PCR. Concording with our previous results, both Cd and E2 *per se* increased global ERα mRNA levels. Co-incubation of cells with Cd plus E2 for 8 h also increased ERα mRNA expression, an effect significantly higher than those observed with Cd or E2 alone, suggesting an additive effect of Cd plus E2 on ERα expression (Figure **8**). 

## Discussion

Several reports have suggested that different environmental contaminants may affect the endocrine system of wildlife species and human life [31]. Many metals can act as endocrine disruptors. In the present study, we showed for the first time that Cd at nanomolar concentrations displayed xenoestrogenic activities by affecting lactotroph proliferation and hormone release from anterior pituitary cells. Cd has been implicated as a cancer causing agent and has been classified by the International Agency for Research on Cancer as a type I human carcinogen [32]. Multiple evidence indicates that this metal is able to act as an endocrine disruptor at micromolar concentrations since it induces cell proliferation in cell lines derived from breast, endometrium and prostate tumors [12,33]. 

Cd has shown a potent estrogen- and androgen-like activity *in vivo* and *in vitro* by directly binding to estrogen and androgen receptors [11]. However, specific mechanisms underlying the effects of Cd as an endocrine disruptor remain to be elucidated. The xenoestrogenic activity of Cd at submicromolar concentrations has been reported in some cell lines, especially tumor-derived cells [7,28]. Nevertheless, our study used primary cultures of anterior pituitary cells and investigated some parameters specifically involved in E2 action on this gland: lactotroph proliferation and prolactin secretion. We also studied Cd effect on ERα expression and its role in Cd E2-like effects. 

Anterior pituitary gland is made up of heterogeneous cell population of which nearly 50% are lactotrophs [34]. Cd effect on proliferation of lactotrophs was observed, but not of somatotrophs nor gonadotrophs. Other hormone-secretory cells (thyrotrophs and corticotrophs) were not studied since their percentage of the population (3%) and renewal rate are very low. Remarkably, the stimulation grade of proliferation caused by 10 nM Cd was analogous to that observed after 1 nM E2 treatment, suggesting that these cells are very sensitive to this metal.

Diverse evidence has shown that Cd at micromolar concentrations as well as other xenoestrogen compounds, are able to stimulate proliferation of several human tumor cell lines such as MCF-7 breast cancer cells [8,12,33] and LNCaP prostate cells [35]. It has also been reported that some xenoestrogens such as endosulphan and chlordane stimulate prolactin secretion without affecting cell growth of the GH3 lactotroph cell line [36]. Our results show that nanomolar concentrations of Cd also promote GH3 cell proliferation, indicating that Cd is able to stimulate proliferation of not only normal but also tumor cells. 

The cell cycle is a finely tuned process whose progression is strictly controlled by the expression and phosphorylation status of specific proteins [37]. Cyclin Ds function as cell cycle regulators modulating progression of cell through the G1/S transition [38]. Other key components in the response to the proliferative signal are the immediate-early c-fos and c-myc genes, whose expression is rapidly induced by mitogenic stimuli [22]. Proliferative E2 signaling through ERα involves up-regulation of many of these genes [23,39]. Cd interaction with a multitude of cell signal transduction pathways, many associated with mitogenic signalling [40], has been widely documented. Multiple evidence, both *in vivo* and *in vitro*, has shown the potential of Cd to mimic estrogen effects at micromolar concentrations in various tissues [6,7,41], and Cd was demonstrated to activate ERα through interaction with the receptor’s hormone-binding domain [6,7,12].

Here, we showed that 10 nM Cd exposure enhanced both Cyc D1 and Cyc D3 expression in an ERα−dependent manner in anterior pituitary cells. Further, c-fos mRNA expression was also increased by Cd treatment. Similar to what was observed in primary cultures, Cd stimulated Cyc D1 and Cyc D3 mRNA expression in GH3 cells. Consequently, it is likely that the increase of cyclins expression occurs mainly in the lactotroph population since these cells showed augmented proliferation after E2 or Cd treatment. 

All together, these results show that Cd, at nanomolar concentration, exerts a potent, ERs-dependent xenoestrogenic effect on cell proliferation of both normal and tumor derivate lactotrophs, indicating that these cells are highly receptive to this metal. 

Prolactin is a well-known estrogen-inducible gene [42]. Previous reports from our laboratory showed that Cd, at micromolar concentrations, is cytotoxic and inhibits prolactin release [26]. However, to our knowledge, no Cd xenoestrogenic effect on this hormone has yet been reported. The present study shows that Cd, at nanomolar concentration, stimulates anterior pituitary prolactin secretion by increasing prolactin mRNA expression and hormone release in ERs-dependent manner. Rousseau et al. reported that nanomolar concentrations of endosulphan, an organochlorine pesticide, as well as chlordane, enhance prolactin mRNA expression in GH3 cells without affecting cell growth [36]. On the contrary, Wade et al. showed that endosulphan does not affect prolactin secretion *in vivo* [43]. Similarly to Cd, another related compound, bisphenol-A, was shown to display potent xenoestrogenic activity by modulating prolactin secretion both *in vivo* and *in vitro* and by stimulating lactotroph proliferation [44]. 

E2 is known to regulate its own receptor expression in different tissues [17]. Here, we demonstrate that Cd was also able to modulate mRNA expression of global ERα in anterior pituitary cells. This expression was increased at 8 and 24 h. On the other hand, Garcia-Morales et al. showed that higher Cd concentrations (1 μM) decreased ERα mRNA levels after 24 h treatment in MCF-7 cells [6]. 

Anterior pituitary cells express ERα protein of 66 kDa (ERα66) and two TERPs, TERP1 and TERP2, corresponding to ERα36 and ERα46, respectively [17]. Previous evidence showed that E2 treatment was able to increase both TERPs’ mRNA levels while full-length ERα and ERβ remained unchanged in anterior pituitary gland from ovariectomized rats. Noticeably, both TERPs’ mRNA levels were found to increase in Fischer 344 rats but only TERP1 was affected in anterior pituitary cells from Sprague-Dawley rats [45]. Our results show that 10 nM Cd, like E2, caused a marked increase of both full-length ERα and TERP2 protein levels. However, our findings do not tally with this previous evidence, mainly due to different experimental designs. Here, we performed our experiments on anterior pituitary cells in culture from Wistar rats at random stages of estrous cycle while Mitchner et al. studied E2 effects on anterior pituitary gland from ovariectomized Fisher 344 and Sprague-Dawley rats. In concordance with our findings, previous data showed that E2 treatment induces mRNA expression of TERP2 in human macrophages in culture [46]. All together, this diverse evidence shows that E2-elicited ERα regulation is cell type-, strain-, and species-specific. 

In the present study we cannot differentiate between ERα mRNA splicing variants since the primers were directed against a common region of all ERα. However, it is likely that the augmented ERα mRNA expression corresponds mainly to an increase in TERPs as revealed after immunoblot analysis. We also observed that Cd and E2 mildly induced ERα full-length expression. To our knowledge, this is the first report to show the up-regulation of ERα66 driven by its natural ligand which is also reproduced by Cd *in vitro*. Since TERPs play a negative role in E2 signaling *per se* and trough binding ERα [47], it is likely that full-length ERα up-regulation does not participate in this pathway. It is also possible that augmented full-length ERα expression is a by-product of ERα mRNA splicing. 

Cd xenoestrogenic effect on MCF-7 cells has been attributed to its ability to activate ERα through interaction with the hormone binding domain of the receptor [7]. Cadmium binds with high affinity in a non-competitive way [48]. Cd interaction with the receptor appeared to involve several amino acids in the hormone-binding pocket of the receptor, suggesting that this metal may form a coordination complex with the hormone binding domain and thereby activate the receptor. Interaction of Cd with ERs involves specifically cysteines 381 and 447, glutamic acid 523, histidine 524, and aspartic acid 538 [7]. Few studies have examined the possibility of an interaction between E2 and different metals or xenoestrogens. Here we show that simultaneous incubation with Cd and E2 had an additive effect on ERα mRNA expression. This result is supported by previous evidence showing that E2 exposure together with some metals as copper, cobalt or lead at micromolar concentrations, results in an additive effect on ERα mRNA and protein expression in MCF-7 cells [8]. 

Here, we showed that potent Cd xenoestrogenic actions take place at subtoxic, nanomolar concentrations in a relative short period of time, which underscores its harmful potential. Dual actions of Cd (proapoptotic or xenoestrogenic) depending on its concentration raise concerns about the impact of this environmental toxin on human health.

In sum, the present work shows for the first time that Cd can display xenoestrogenic activities by inducing cell growth and stimulating prolactin secretion from anterior pituitary cells. Considering that prolactin is involved in the regulation of many physiological processes such as growth, development, metabolism and reproduction, the disruption of prolactin secretion by Cd can play a pivotal role in fertility and reproductive disorders associated with Cd contamination. Our results support the hypothesis that, as a xenoestrogenic agent, Cd can disrupt hormone status thus affecting reproductive functions. Even further, Cd E2-like actions can be of major importance in neoplastic processes in estrogen-dependent tissues. 

## References

[1] Kah M, Levy L, Brown C (2012) Potential for effects of land contamination on human health. The case of cadmium. J Toxicol Environ Health 15: 348-363. doi:10.1080/10937404.2012.705107. 22852814

[2] Cabrera C, Ortega E, Lorenzo ML, López MC (1998) Cadmium contamination of vegetable crops, farmlands, and irrigation waters. Rev Environ Contam Toxicol 154: 55-81. PubMed: 9414631. 9414631 10.1007/978-1-4612-2208-8_2

[3] Poliandri AH, Machiavelli LI, Quinteros AF, Cabilla JP, Duvilanski BH (2006) Nitric oxide protects the mitochondria of anterior pituitary cells and prevents cadmium-induced cell death by reducing oxidative stress. Free Radic Biol Med 40: 679–688. doi:10.1016/j.freeradbiomed.2005.09.021. PubMed: 16458199. 16458199

[4] Carlsen E, Giwercman A, Keiding N, Skakkebaek NE (1992) Evidence for decreasing quality of semen during past 50 years. BMJ 305: 609-613. doi:10.1136/bmj.305.6854.609. PubMed: 1393072. 1393072 PMC1883354

[5] Diamanti-Kandarakis E, Bourguignon JP, Giudice LC, Hauser R, Prins GS et al. (2009) Endocrine-disrupting chemicals: an Endocrine Society scientific statement. Endocr Rev 30: 293-342. doi:10.1210/er.2009-0002. PubMed: 19502515. 19502515 PMC2726844

[6] Garcia-Morales P, Saceda M, Kenney N, Kim N, Salomon DS et al. (1994) Effect of cadmium on estrogen receptor levels and estrogen-induced responses in human breast cancer cells. J Biol Chem 269: 16896-16901. PubMed: 8207012. 8207012

[7] Stoica A, Katzenellenbogen BS, Martin MB (2000) Activation of estrogen receptor-alpha by the heavy metal cadmium. Mol Endocrinol 14: 545-553. doi:10.1210/me.14.4.545. PubMed: 10770491. 10770491

[8] Martin MB, Reiter R, Pham T, Avellanet YR, Camara J et al. (2003) Estrogen-like activity of metals in MCF-7 breast cancer cells. Endocrinology 144: 2425–2436. doi:10.1210/en.2002-221054. PubMed: 12746304. 12746304

[9] Aquino NB, Sevigny MB, Sabangan J, Louie MC (2012) The role of cadmium and nickel in estrogen receptor signaling and breast cancer: metalloestrogens or not? J Environ Sci Health C Environ Carcinog Ecotoxicol Rev 30: 189-224. doi:10.1080/10590501.2012.705159. PubMed: 22970719. 22970719 PMC3476837

[10] Johnson MD, Kenney N, Stoica A, Hilakivi-Clarke L, Singh B et al. (2003) Cadmium mimics the in vivo effects of estrogen in the uterus and mammary gland. Nat Med 9: 1081–1084. doi:10.1038/nm902. PubMed: 12858169. 12858169

[11] Takiguchi M, Yoshihara S (2006) New aspects of cadmium as endocrine disruptor. J Environ Sci 13: 107-116. PubMed: 16788562. 16788562

[12] Byrne C, Divekar SD, Storchan GB, Parodi DA, Martin MB (2009) Cadmium - a metallohormone? Toxicol Appl Pharmacol 238: 266–271. doi:10.1016/j.taap.2009.03.025. PubMed: 19362102. 19362102 PMC2709711

[13] Spady TJ, McComb RD, Shull JD (1999) Estrogen action in the regulation of cell proliferation, cell survival, and tumorigenesis in the rat anterior pituitary gland. Endocrine 11: 217-233. doi:10.1385/ENDO:11:3:217. PubMed: 10786818. 10786818

[14] Zárate S, Jaita G, Zaldivar V, Radl DB, Eijo G et al. (2009) Estrogens exert a rapid apoptotic action in anterior pituitary cells. Am J Physiol Endocrinol Metab 296: 664-671. doi:10.1152/ajpendo.90785.2008. PubMed: 19158323. 19158323

[15] Enmark E, Gustafsson JA (1999) Oestrogen receptors - an overview. J Intern Med 246: 133-138. doi:10.1046/j.1365-2796.1999.00545.x. PubMed: 10447781. 10447781

[16] Shanle EK, Xu W (2011) Endocrine disrupting chemicals targeting estrogen receptor signaling: Identification and mechanisms of action. Chem Res Toxicol 24: 6-19. doi:10.1021/tx100231n. PubMed: 21053929. 21053929 PMC3119362

[17] Shupnik MA (2002) Oestrogen receptors, receptor variants and oestrogen actions in the hypothalamic –pituitary axis. J Neuroendocrinol 14: 85-94. doi:10.1046/j.0007-1331.2001.00744.x. PubMed: 11849367. 11849367

[18] Taylor SE, Martin-Hirsch PL, Martin FL (2010) Oestrogen receptor splice variants in the pathogenesis of disease. Cancer Lett 288: 133-148. doi:10.1016/j.canlet.2009.06.017. PubMed: 19608332. 19608332

[19] Seilicovich A (2010) Cell life and death in the anterior pituitary gland: role of oestrogens. J Neuroendocrinol 22: 758-764. PubMed: 20456596. 20456596 10.1111/j.1365-2826.2010.02010.x

[20] Sánchez I, Dynlacht BD (2005) New insights into cyclins, CDKs, and cell cycle control. Semin Cell Dev Biol 16: 311-321. doi:10.1016/j.semcdb.2005.02.007. PubMed: 15840440. 15840440

[21] Butt AJ, McNeil CM, Musgrove EA, Sutherland RL (2005) Downstream targets of growth factor and oestrogen signalling and endocrine resistance: the potential roles of c-Myc, cyclin D1 and cyclin E. Endocr Relat Cancer 12: 47-59. doi:10.1677/erc.1.00993. PubMed: 16113099. 16113099

[22] Shaulian E, Karin M (2002) AP-1 as a regulator of cell life and death. Nat Cell Biol 4: 131-136. doi:10.1038/ncb0502-e131. PubMed: 11988758. 11988758

[23] Allen DL, Mitchner NA, Uveges TE, Nephew KP, Khan S et al. (1997) Cell-specific induction of c-fos expression in the pituitary gland by estrogen. Endocrinology 138: 2128-2135. doi:10.1210/en.138.5.2128. PubMed: 9112413. 9112413

[24] Lafuente A, Esquifino AI (1999) Cadmium effects on hypothalamic activity and pituitary hormone secretion in the male. Toxicol Lett 110: 209-218. doi:10.1016/S0378-4274(99)00159-9. PubMed: 10597030. 10597030

[25] Siu ER, Mruk DD, Porto CS, Cheng CY (2009) Cadmium-induced testicular injury. Toxicol Appl Pharmacol 238: 240-249. doi:10.1016/j.taap.2009.01.028. PubMed: 19236889. 19236889 PMC2804910

[26] Poliandri AH, Cabilla JP, Velardez MO, Bodo CC, Duvilanski BH (2003) Cadmium induces apoptosis in anterior pituitary cells that can be reversed by treatment with antioxidants. Toxicol Appl Pharmacol 190: 17-24. doi:10.1016/S0041-008X(03)00191-1. PubMed: 12831779. 12831779

[27] Miler EA, Nudler SI, Quinteros FA, Cabilla JP, Ronchetti SA et al. (2010) Cadmium-induced oxidative stress in pituitary gland is reversed by removing the contamination source. Hum Exp Toxicol 29: 873-880. doi:10.1177/0960327110362703. PubMed: 20197452. 20197452

[28] Yu X, Filardo EJ, Shaikh ZA (2010) The membrane estrogen receptor GPR30 mediates cadmium-induced proliferation of breast cancer cells. Toxicol Appl Pharmacol 245: 83-90. doi:10.1016/j.taap.2010.02.005. PubMed: 20153348. 20153348

[29] Velardez MO, Benitez AH, Cabilla JP, Bodo CC, Duvilanski BH (2003) Nitric oxide decreases the production of inositol phosphates stimulated by angiotensin II and thyrotropin-releasing hormone in anterior pituitary cells. Eur J Endocrinol 148: 89-97. doi:10.1530/eje.0.1480089. PubMed: 12534362. 12534362

[30] Niswender GD, Chen CL, Midgley AR Jr, Meites J, Ellis S (1969) Radioimmunoassay for rat prolactin. Proc Soc Exp Biol Med 130: 793–797. doi:10.3181/00379727-130-33657. PubMed: 5813056. 5813056

[31] De Coster S, van Larebeke N (2012) Endocrine-disrupting chemicals: associated disorders and mechanisms of action. J Environ Public Health 2012:713696. PubMed: 22991565 10.1155/2012/713696 PMC3443608 22991565

[32] Waisberg M, Joseph P, Hale B, Beyersmann D (2003) Molecular and cellular mechanisms of cadmium carcinogenesis. Toxicology 192: 95-117. doi:10.1016/S0300-483X(03)00305-6. PubMed: 14580780. 14580780

[33] Siewit CL, Gengler B, Vegas E, Puckett R, Louie MC (2010) Cadmium promotes breast cancer cell proliferation by potentiating the interaction between ER alpha and c-Jun. Mol Endocrinol 24: 981–992. doi:10.1210/me.2009-0410. PubMed: 20219890. 20219890 PMC2870938

[34] Chen HT (1987) Postnatal development of pituitary lactotropes in the rat measured by reverse hemolytic plaque assay. Endocrinology 120: 247-253. doi:10.1210/endo-120-1-247. PubMed: 3780561. 3780561

[35] Martin MB, Voeller HJ, Gelmann EP, Lu J, Stoica EG et al. (2002) Role of cadmium in the regulation of AR gene expression and activity. Endocrinology 143: 263-275. doi:10.1210/en.143.1.263. PubMed: 11751618. 11751618

[36] Rousseau J, Cossette L, Grenier S, Martinoli MG (2002) Modulation of prolactin expression by xenoestrogens. Gen Comp Endocrinol 126: 175-182. doi:10.1006/gcen.2002.7789. PubMed: 12030773. 12030773

[37] Montanari M, Macaluso M, Cittadini A, Giordano A (2006) Role of geminin: from normal control of DNA replication to cancer formation and progression? Cell Death Differ 13: 1052-1056. doi:10.1038/sj.cdd.4401932. PubMed: 16628231. 16628231

[38] Qian X, Kulig E, Jin L, Lloyd RV (1998) Expression of D-type cyclins in normal and neoplastic rat pituitary. Endocrinology 139: 2058-2067. doi:10.1210/en.139.4.2058. PubMed: 9528994. 9528994

[39] Teng J, Wang ZY, Jarrard DF, Bjorling DE (2008) Roles of estrogen receptor alpha and beta in modulating urothelial cell proliferation. Endocr Relat Cancer 15: 351-364. doi:10.1677/erc.1.01255. PubMed: 18310301. 18310301 PMC3513362

[40] Brama M, Gnessi L, Basciani S, Cerulli N, Politi L et al. (2007) Cadmium induces mitogenic signaling in breast cancer cell by an ERalpha-dependent mechanism. Mol Cell Endocrinol 264: 102-108. doi:10.1016/j.mce.2006.10.013. PubMed: 17125913. 17125913

[41] Höfer N, Diel P, Wittsiepe J, Wilhelm M, Kluxen FM et al. (2010) Investigations on the estrogenic activity of the metallohormone cadmium in the rat intestine. Arch Toxicol 84: 541–552. doi:10.1007/s00204-010-0524-x. PubMed: 20186393. 20186393

[42] Maurer RA, Kim KE, Day RN, Notides AC (1990) Regulation of prolactin gene expression by estradiol. Molecular Endocrinology and Steroid Hormone Action. New York: Liss pp. 159–169. 2406729

[43] Wade MG, Desaulniers D, Leingartner K, Foster WG (1997) Interactions between endosulfan and dieldrin on estrogen-mediated processes in vitro and in vivo. Reprod Toxicol 11: 791–798. doi:10.1016/S0890-6238(97)00062-2. PubMed: 9407589. 9407589

[44] Steinmetz R, Brown NG, Allen DL, Bigsby RM, Ben-Jonathan N (1997) The environmental estrogen bisphenol A stimulates prolactin release in vitro and in vivo. Endocrinology 138: 1780-1786. doi:10.1210/en.138.5.1780. PubMed: 9112368. 9112368

[45] Mitchner NA, Garlick C, Ben-Jonathan N (1998) Cellular distribution and gene regulation of estrogen receptors alpha and beta in the rat pituitary gland. Endocrinology 139: 3976-3983. doi:10.1210/en.139.9.3976. PubMed: 9724053. 9724053

[46] Murphy AJ, Guyre PM, Wira CR, Pioli PA (2009) Estradiol regulates expression of estrogen receptor ERα46 in human macrophages. PLOS ONE 4: 5539. doi:10.1371/journal.pone.0005539. PMC2678254 19440537

[47] Denger S, Reid G, Kos M, Flouriot G, Parsch D et al. (2001) ERalpha gene expression in human primary osteoblasts: evidence for the expression of two receptor proteins. Mol Endocrinol 15: 2064–2077. doi:10.1210/me.15.12.2064. PubMed: 11731609. 11731609

[48] Fechner P, Damdimopoulou P, Gauglitz G (2011) Biosensors paving the way to understanding the interaction between cadmium and the estrogen receptor alpha. PLOS ONE 6: 23048. doi:10.1371/journal.pone.0023048. PubMed: 21829690. PMC3149063 21829690

